# MRI-Based Patterns of Meniscal Tears and Associated Intra-Articular Abnormalities in Patients at a Tertiary Care Center in Riyadh, Saudi Arabia: A Retrospective Cross-Sectional Study

**DOI:** 10.3390/diagnostics16213650

**Published:** 2026-11-04

**Authors:** Haniyya Al-Rehaily, Abdullah Al-Zahrani, Badar Aboshaiqah, Naif Alharbi, Ahmad M. Rayani, Ahmad Aboshaiqah

**Affiliations:** 1 Medical Imaging Department, King Abdulaziz Medical City, Ministry of National Guard Health Affairs, Riyadh 11426, Saudi Arabia; dr.haniyya@hotmail.com (H.A.-R.); nzeh.07@gmail.com (A.A.-Z.); b.aboshaiqah@gmail.com (B.A.); 2 King Abdullah International Medical Research Center (KAIMRC), Riyadh 11481, Saudi Arabia; 3 College of Medicine, King Saud Bin Abdulaziz University for Health Sciences, Riyadh 11481, Saudi Arabia; 4 Community and Psychiatric Mental Health Nursing Department, College of Nursing, King Saud University, Riyadh 12372, Saudi Arabia; arayani@ksu.edu.sa; 5 Department of Nursing Administration and Education, College of Nursing, King Saud University, Riyadh 12372, Saudi Arabia; aaboshaiqah@ksu.edu.sa

**Keywords:** meniscus, meniscal tear, magnetic resonance imaging, tear morphology, knee injuries, anterior cruciate ligament, degenerative tear, traumatic tear, Saudi Arabia

## Abstract

**Background**: Meniscal tears are common intra-articular knee injuries that arise from acute trauma or degenerative change and frequently coexist with ligamentous and chondral abnormalities. Magnetic resonance imaging (MRI) allows noninvasive characterization of tear location, morphology, and associated pathology, but MRI-based data from Saudi Arabia are scarce. **Objective**: To describe the MRI patterns of meniscal tears and associated intra-articular abnormalities and their distribution by age, sex, and mechanism of injury, in patients evaluated at a tertiary care center in Riyadh, Saudi Arabia. **Methods**: In this retrospective cross-sectional study, consecutive patients aged ≥15 years with meniscal tears reported on knee MRI at King Abdulaziz Medical City, Riyadh, between January 2020 and December 2023 were included. Data were extracted from original radiology reports and electronic medical records. Groups were compared using chi-square or Fisher’s exact tests; unadjusted odds ratios (ORs) with 95% confidence intervals (CIs) were calculated, and *p*-values were adjusted using the Benjamini–Hochberg false discovery rate (FDR). **Results**: Of 496 patients (65.7% male; mean age 41.8 ± 13.2 years), 60.3% had a traumatic mechanism. The medial meniscus was involved in 415 patients (83.7%; isolated in 69.4%). Horizontal tears were the most frequently reported pattern (27.2%), followed by radial (19.6%) and root (19.4%) tears. Joint effusion (58.7%), anterior cruciate ligament (ACL) tear (36.1%), and cartilage loss (34.3%) were the most frequent associated findings. Traumatic cases were younger (median 34 vs. 51 years) and more often had isolated lateral meniscal, horizontal, vertical, bucket-handle, and complex tears and ACL tears (49.2% vs. 16.2%; OR 4.99, 95% CI 3.21–7.75), whereas degenerative cases more often had radial, root, and degenerative-pattern tears, cartilage loss (61.9% vs. 16.1%), and joint effusion. These associations remained significant after FDR adjustment. Surgical management was recorded in 67.1% of patients. **Conclusions**: In this hospital-based sample, meniscal tear patterns and associated MRI findings differed by age, sex, and mechanism of injury. Because the data derive from a single referral center and from original clinical reports, the findings describe a selected population, should not be extrapolated to the general Saudi population, and are hypothesis-generating.

## 1. Introduction

Meniscal tears are among the most common intra-articular injuries of the knee and one of the most frequent reasons for orthopedic referral and knee magnetic resonance imaging (MRI) [1,2]. In hospital-based epidemiological series, the incidence of meniscal injury has been estimated at approximately 0.7 per 1000 inhabitants per year, with a male predominance and a peak in the third to fifth decades of life [3,4]. The menisci transmit and distribute axial load, absorb shock, and contribute to joint congruity and stability; disruption of their circumferential collagen architecture impairs hoop-stress transmission, increases tibiofemoral contact pressure, and predisposes the knee to cartilage degeneration and osteoarthritis [5,6,7]. Because only the peripheral 10–25% of the adult meniscus is vascularized, tear location also determines healing potential and the feasibility of repair [8].

Meniscal tears are broadly categorized as traumatic or degenerative. Traumatic tears follow an identifiable injury with an acute onset of symptoms, typically a twisting or compressive load in younger, physically active individuals, and frequently accompany anterior cruciate ligament (ACL) rupture [9,10]. Degenerative lesions develop gradually, without significant trauma, in middle-aged and older individuals, often as horizontal cleavage, complex, or root tears; they frequently coexist with chondral loss and osteoarthritis and are common incidental findings on MRI in asymptomatic older adults [11,12,13]. The medial meniscus is injured more often than the lateral meniscus, a difference attributed to its firmer capsular and ligamentous fixation and lower mobility, whereas the more mobile lateral meniscus is relatively more often involved in acute injuries in younger patients, particularly in association with ACL tears [10,14,15,16]. MRI is the noninvasive reference modality for evaluating suspected meniscal injury. Compared with arthroscopy, it shows high diagnostic performance, with pooled sensitivities of approximately 91% for medial and 79% for lateral meniscal tears [17,18], and it allows description of tear location and morphology together with concomitant ligamentous, chondral, and osseous abnormalities that influence management [19,20,21].

Despite the clinical burden of knee injuries in Saudi Arabia, published local MRI data on meniscal tears are limited. Available studies have mainly evaluated the diagnostic accuracy of MRI against arthroscopy in small surgical samples [22,23], described associated injuries only among patients with ACL tears [24], or assessed osteoarthritis rather than meniscal pathology [25]. To our knowledge, no study from the region has jointly described tear location, tear morphology, associated intra-articular abnormalities, and recorded management in a large consecutive MRI-based sample stratified by age, sex, and mechanism of injury, with effect sizes and correction for multiple testing. Such data provide a local reference for radiologists and orthopedic surgeons interpreting knee MRI, support service planning, and identify patterns that differ from published series and therefore merit prospective study. The aim of this study was to describe the MRI patterns of meniscal tears and associated intra-articular abnormalities in patients evaluated at a tertiary care center in Riyadh, Saudi Arabia, and to examine their distribution according to age, sex, and mechanism of injury.

## 2. Materials and Methods

### 2.1. Study Design and Reporting

This was a retrospective, single-center, cross-sectional study based on the review of radiology reports and electronic medical records. Each patient contributed a single index MRI examination, and no longitudinal follow-up was undertaken; the design is therefore cross-sectional rather than cohort. The study is reported in accordance with the Strengthening the Reporting of Observational Studies in Epidemiology (STROBE) guideline for cross-sectional studies [26], and the completed checklist is provided as Supplementary Table S1.

### 2.2. Setting

The study was conducted in the Department of Medical Imaging at King Abdulaziz Medical City (KAMC), Ministry of National Guard Health Affairs (MNGHA), Riyadh, Saudi Arabia, which maintains a comprehensive radiological database of MRI examinations. KAMC is a tertiary-care academic referral center and Level I trauma center that serves National Guard personnel, their dependents, and eligible patients referred from affiliated primary and secondary care facilities. The department performs approximately 1600–800 knee MRI examinations per year, requested from outpatient orthopedic and sports medicine clinics, the emergency department, and inpatient services. The study period (January 2020 to December 2023) included the COVID-19 pandemic, during which elective outpatient services in Saudi Arabia were temporarily restricted in 2020; no other major changes in referral pathways, clinical practice, or the institutional knee MRI protocol occurred during the study period.

### 2.3. Participants

**Figure 1:**
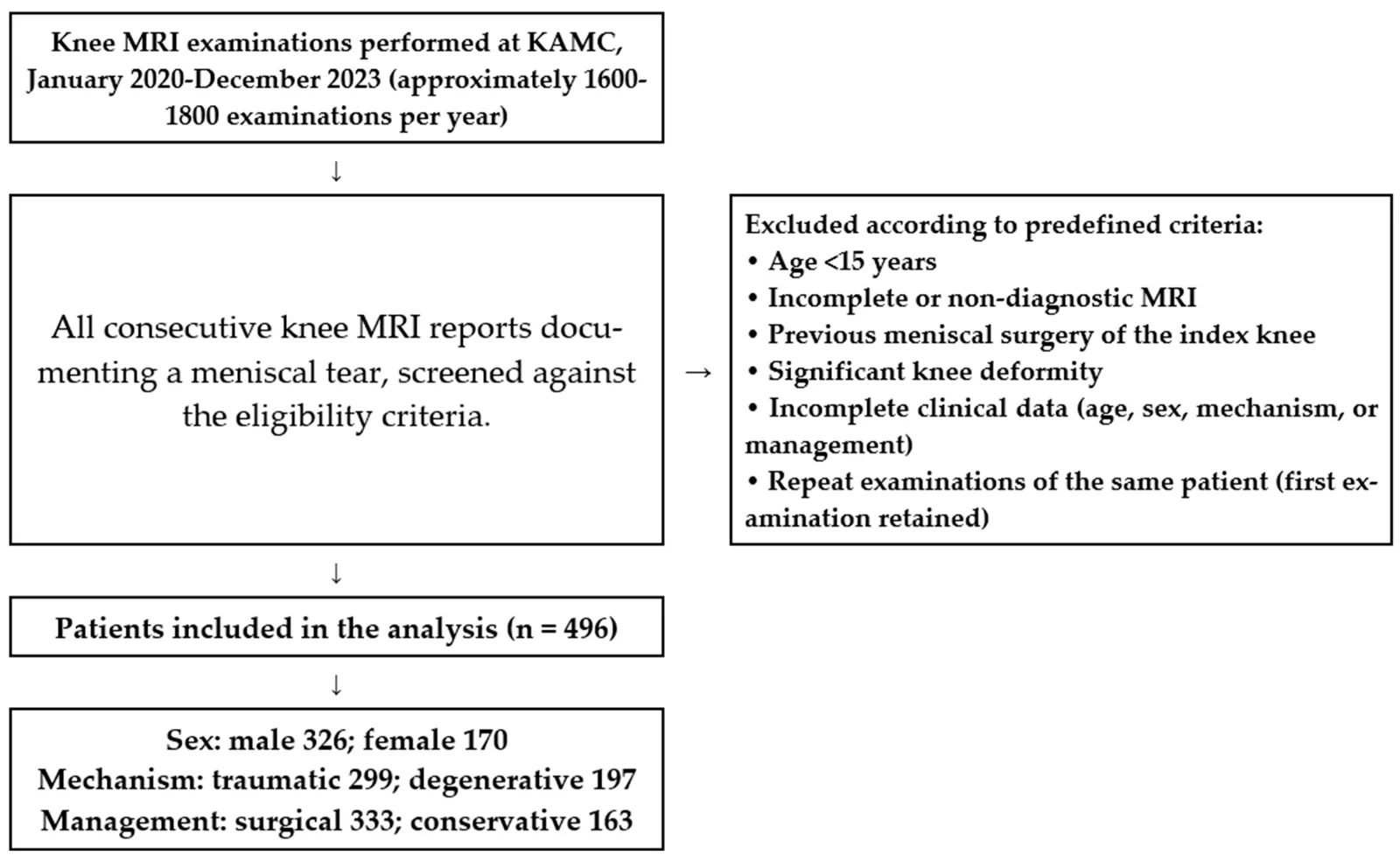
Flow diagram of patient selection (STROBE). The predefined exclusion criteria were applied to all consecutive knee MRI reports documenting a meniscal tear during the study period; the final analytic sample comprised 496 patients. KAMC, King Abdulaziz Medical City; MRI, magnetic resonance imaging.

All consecutive patients who underwent knee MRI between 1 January 2020 and 31 December 2023 and whose MRI report documented a meniscal tear were identified through the radiology information system (RIS) and picture archiving and communication system (PACS) and screened for eligibility. Inclusion criteria were (i) age ≥ 15 years at the time of MRI; (ii) a meniscal tear reported on MRI; and (iii) availability of complete MRI and relevant clinical data (age, sex, documented mechanism of injury, and management). Both male and female patients were included. Patients younger than 15 years were excluded because developmental differences in meniscal vascularity and tissue properties influence tear patterns and healing potential [8]. Other exclusion criteria were incomplete or poor-quality (non-diagnostic) MRI examinations, a history of previous meniscal surgery of the index knee, or significant knee deformity that could interfere with accurate image interpretation. When a patient had more than one eligible examination, only the first examination during the study period was included. The selection process is summarized in Figure 1.

### 2.4. MRI Acquisition

All MRI examinations were acquired on a 3-T system (MAGNETOM Vida, Siemens Healthineers, Erlangen, Germany) with a dedicated knee coil, using the institutional routine knee protocol. Multiplanar imaging was acquired in the axial, coronal, and sagittal planes, and the standard musculoskeletal protocol included T1-weighted, T2-weighted, and proton density (PD)-weighted fat-suppressed sequences, with a slice thickness of 3–4 mm, an interslice gap of 0.3–0.4 mm, a field of view of 14–16 cm, and an in-plane matrix of approximately 320 × 256. The same scanner and core sequences were used throughout the study period.

### 2.5. Image Interpretation and Data Extraction

This study was based on the original clinical MRI reports; the images were not re-read for research purposes. Reports were issued during routine practice by consultant radiologists, including fellowship-trained musculoskeletal radiologists, who had access to the clinical information provided on the imaging request, as is standard in clinical reporting; the readers were therefore not blinded to the clinical history. A meniscal tear was diagnosed using standard MRI criteria, namely intrameniscal signal unequivocally extending to an articular surface and/or distortion of normal meniscal morphology [17,19,27]. Data were extracted by two investigators (H.A.-R. and A.A.-Z.) using a standardized data collection form, and ambiguous entries were resolved by consensus with a senior musculoskeletal radiologist (N.A.).

### 2.6. Variables and Definitions

Tear location was categorized as isolated medial meniscus, isolated lateral meniscus, or both menisci. Tear pattern was recorded from the report wording using seven descriptive categories: horizontal (cleavage plane parallel to the tibial plateau); vertical (longitudinal tear perpendicular to the tibial plateau and parallel to the circumferential fibers, without displacement); bucket-handle (displaced vertical longitudinal tear with the inner fragment displaced into the intercondylar notch); radial (perpendicular to both the tibial plateau and the circumferential fibers); root (tear or avulsion within 1 cm of the meniscal root attachment); complex traumatic (tear in two or more planes reported in the setting of acute injury); and degenerative (tear described as degenerative, typically with irregular or macerated margins and intrasubstance degeneration, without a single dominant plane) [19,20]. These categories are reporting descriptors rather than a formal classification and are not mutually exclusive: “complex traumatic” and “degenerative” combine morphological and etiological information, and more than one pattern could be reported in the same knee. When a report specified a more specific descriptor (e.g., a radial tear at the posterior root or a displaced longitudinal tear), the more specific category (root; bucket-handle) was recorded. Each pattern was therefore analyzed as a separate binary variable (present/absent), and the total number of patterns (*n* = 547) exceeds the number of patients (*n* = 496). These categories do not correspond directly to the arthroscopic International Society of Arthroscopy, Knee Surgery and Orthopaedic Sports Medicine (ISAKOS) classification [28], which could not be applied retrospectively to report-based data.

The mechanism of injury was classified from the clinical history documented on the imaging request and in the medical record. In line with the European Society of Sports Traumatology, Knee Surgery and Arthroscopy (ESSKA) consensus definitions [9,11], a tear was classified as traumatic when an acute onset of symptoms followed an identifiable injury (e.g., sports or twisting injury, fall, or road traffic accident) and as degenerative when symptoms developed gradually without significant trauma. When the documented history was insufficient, the mechanism indicated by the reporting radiologist, which could incorporate imaging features, was used. Consequently, the mechanism variable is not fully independent of MRI morphology, and this was considered when interpreting the mechanism-stratified analyses.

Associated intra-articular abnormalities comprised anterior cruciate ligament (ACL), posterior cruciate ligament (PCL), medial collateral ligament (MCL), and lateral collateral ligament (LCL) tears; cartilage loss (focal or diffuse chondral thinning or defect); bone contusion (bone marrow edema pattern reported as contusion); and joint effusion. Patients with none of these findings were categorized as “none”, and more than one finding could be present in the same patient.

Management was recorded from the electronic medical record as a binary patient-level variable. Surgical management was defined as any arthroscopic or open procedure on the index knee documented after the index MRI (e.g., meniscal repair, partial meniscectomy, or ligament reconstruction), whether performed for the meniscal tear or for an associated pathology. Conservative management was defined as the absence of a documented surgical procedure (e.g., physiotherapy, analgesia, activity modification, or bracing). Management was ascertained from the medical records available at the time of data extraction. The specific procedure, its indication, and its timing were not captured.

### 2.7. Statistical Analysis

The collected data were exported into Microsoft Excel and analyzed using the Statistical Package for the Social Sciences (SPSS) version 26.0 (IBM-SPSS, Armonk, New York, NY, USA). Descriptive statistics were used to summarize categorical variables as frequencies and percentages. Numerical variables were described as the mean and standard deviation (SD) for normally distributed data, and as the median and interquartile range (IQR) for non-normally distributed data, based on the results of the Shapiro-Wilk test. For overall prevalence estimates, 95% confidence intervals (CIs) were calculated using the Wilson method. The association between categorical variables was assessed using the Pearson chi-square test or Fisher’s exact test (when more than 20% of expected cell counts were below five). The Mann–Whitney U test was used to compare medians (IQR) between two independent groups (males vs. females and traumatic vs. degenerative cases); age was not compared across age groups because age group was the stratifying variable. Effect sizes were reported as unadjusted odds ratios (ORs) with 95% CIs (Woolf method) for two-group comparisons and as Cramér’s V for comparisons across age groups; ORs were not estimated when a cell contained zero. To account for multiple comparisons, *p*-values within each table were adjusted using the Benjamini–Hochberg false discovery rate (FDR) procedure [29], and adjusted values (q) are reported alongside unadjusted *p*-values. Because the analyses were univariable, associations were interpreted as descriptive and potentially confounded, particularly by age. A two-sided *p* < 0.05 (and q < 0.05 after FDR adjustment) was considered statistically significant. Complete data were an inclusion criterion; therefore, there were no missing data for the analyzed variables.

## 3. Results

### 3.1. Participant Characteristics

Patient selection is summarized in Figure 1. A total of 496 patients were included, comprising 326 (65.7%) males and 170 (34.3%) females, with a mean age of 41.8 ± 13.2 years (median 40 years; range 15–87 years); 360 patients (72.6%) were aged 26–55 years. A traumatic mechanism was documented in 299 patients (60.3%). The tear involved the medial meniscus alone in 344 patients (69.4%), the lateral meniscus alone in 81 (16.3%), and both menisci in 71 (14.3%); overall, the medial meniscus was involved in 415 patients (83.7%) and the lateral meniscus in 152 (30.6%). A total of 547 tear patterns were recorded in 496 patients. Horizontal tears were the most frequent pattern (*n* = 135; 27.2%), followed by radial (19.6%), root (19.4%), degenerative (18.1%), complex traumatic (15.5%), vertical (5.6%), and bucket-handle (4.8%) tears. The most frequent associated findings were joint effusion (58.7%), ACL tear (36.1%), cartilage loss (34.3%), and bone contusion (25.8%); 85 patients (17.1%) had no associated abnormality. Surgical management was recorded in 333 patients (67.1%) (Table 1).

### 3.2. Differences by Sex

Females were older than males (median 49 vs. 36 years, *p* < 0.001), and a traumatic mechanism was more frequent in males (73.6% vs. 34.7%; OR 5.25, 95% CI 3.52–7.84). The distribution of tear location differed by sex (*p* = 0.017, q = 0.034; Cramér’s V = 0.13): isolated medial meniscal tears were more frequent in females (77.1% vs. 65.3%), whereas isolated lateral meniscal tears were more frequent in males (19.3% vs. 10.6%). Radial (30.0% vs. 14.1%), root (24.7% vs. 16.6%), and degenerative (23.5% vs. 15.3%) tears were more frequent in females, whereas bucket-handle (7.4% vs. 0%), complex traumatic (20.9% vs. 5.3%), and vertical (7.4% vs. 2.4%) tears were more frequent in males. Males more often had ACL tears (48.8% vs. 11.8%; OR 7.14, 95% CI 4.27–11.95), whereas females more often had cartilage loss (57.1% vs. 22.4%; OR for males 0.22, 95% CI 0.15–0.32). PCL tears were uncommon (0.6% vs. 2.9%), and the difference did not reach statistical significance (Fisher’s exact *p* = 0.050; q = 0.071). Surgical management was more frequent in females (86.5% vs. 57.1%; *p* < 0.001). All other statistically significant differences remained significant after FDR adjustment (Table 2). Because females were substantially older and more often had a degenerative mechanism, these sex differences are likely to be confounded by age.

### 3.3. Differences by Age Group

**Figure 2:**
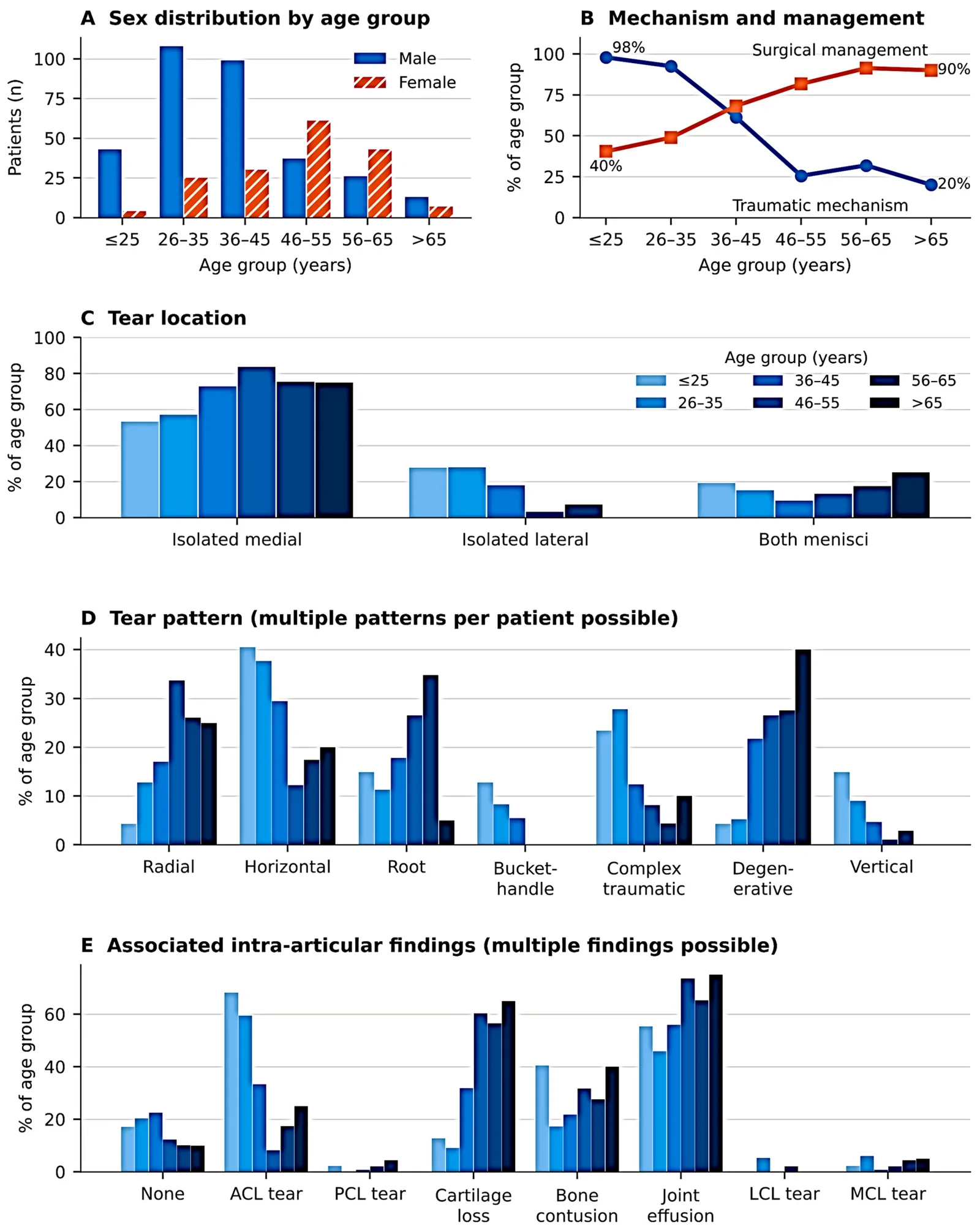
Distribution of demographic and clinical characteristics by age group. (**A**) Number of male and female patients; (**B**) proportion of patients with a traumatic mechanism and proportion managed surgically; (**C**) tear location; (**D**) tear patterns; (**E**) associated intra-articular findings. Panels (**B**–**E**) show percentages of each age group (denominators: ≤25 years, *n* = 47; 26–35 years, *n* = 133; 36–45 years, *n* = 129; 46–55 years, *n* = 98; 56–65 years, *n* = 69; >65 years, *n* = 20). Tear patterns and associated findings are multiple-response variables. ACL, anterior cruciate ligament; LCL, lateral collateral ligament; MCL, medial collateral ligament; PCL, posterior cruciate ligament.

The proportion of traumatic injuries decreased from 97.9% in patients aged ≤25 years to 20.0% in those aged >65 years (*p* < 0.001; V = 0.59). Isolated lateral meniscal tears were most frequent in patients aged ≤35 years (27.7–27.8%) and uncommon in those aged ≥46 years (0–7.2%), whereas isolated medial tears were more frequent in older patients (*p* < 0.001). Radial and degenerative tears were more frequent in patients aged ≥46 years, and root tears peaked at 46–65 years, whereas horizontal, bucket-handle, complex traumatic, and vertical tears were more frequent in younger age groups (all q ≤ 0.011). ACL tears were most frequent in patients aged ≤25 years (68.1%) and 26–35 years (59.4%), whereas cartilage loss increased from 9.0–12.8% in patients aged ≤35 years to 56.5–65.0% in those aged ≥46 years (both *p* < 0.001). Joint effusion was more frequent in patients aged ≥46 years (65.2–75.0%) than in younger patients (45.9–55.8%; *p* = 0.001), and bone contusion showed a non-linear distribution, being most frequent in the youngest (40.4%) and oldest (40.0%) groups (*p* = 0.009). LCL tears were rare and concentrated in the 26–35-year group (*p* = 0.030). The proportion of surgically managed patients increased from 40.4% in patients aged ≤25 years to 90.0–91.3% in those aged >55 years (*p* < 0.001). All statistically significant differences remained significant after FDR adjustment (Table 3 and Figure 2).

### 3.4. Differences by Mechanism of Injury

Patients with traumatic injuries were younger than those with degenerative injuries (median 34 vs. 51 years, *p* < 0.001) and were more often male (80.3% vs. 43.7%). Tear location differed by mechanism (*p* < 0.001; V = 0.21): isolated lateral meniscal tears were more frequent in traumatic cases (22.7% vs. 6.6%), whereas isolated medial tears were more frequent in degenerative cases (77.7% vs. 63.9%). Horizontal (37.8% vs. 11.2%; OR 4.83, 95% CI 2.93–7.98), bucket-handle (7.7% vs. 0.5%), complex traumatic (25.8% vs. 0%), and vertical (9.4% vs. 0%) tears were more frequent in traumatic cases, whereas radial (29.9% vs. 12.7%), root (24.9% vs. 15.7%; *p* = 0.012), and degenerative (43.7% vs. 1.3%) tears were more frequent in degenerative cases. ACL tears (49.2% vs. 16.2%; OR 4.99, 95% CI 3.21–7.75), MCL tears (4.7% vs. 1.0%; *p* = 0.029), and the absence of any associated abnormality (20.7% vs. 11.7%; *p* = 0.009) were more frequent in traumatic cases, whereas cartilage loss (61.9% vs. 16.1%), joint effusion (67.0% vs. 53.2%; *p* = 0.002), and PCL tears (3.0% vs. 0.3%; *p* = 0.017) were more frequent in degenerative cases. Bone contusion did not differ significantly (23.4% vs. 29.4%; *p* = 0.133). Surgical management was recorded in 86.3% of degenerative and 54.5% of traumatic cases (*p* < 0.001). All statistically significant associations persisted after FDR adjustment (Table 4). The complete or near-complete separation of the complex traumatic, vertical, and degenerative categories by mechanism reflects, at least in part, the etiological content of these descriptors (Section 2.6).

### 3.5. Management

The proportion of patients managed surgically was highest among those with degenerative tears (90.0%; OR 5.50, 95% CI 2.68–11.27), cartilage loss (81.8%; OR 3.05, 95% CI 1.95–4.77), and radial tears (77.3%; OR 1.86, 95% CI 1.11–3.13), and lowest among those with bucket-handle tears (16.7%; OR 0.09, 95% CI 0.03–0.26), complex traumatic tears (42.9%; OR 0.30, 95% CI 0.18–0.49), and ACL tears (43.0%; OR 0.18, 95% CI 0.12–0.27). The associations for radial tears (q = 0.043) and for the absence of associated abnormalities (77.6% surgical; q = 0.049) were of borderline significance after FDR adjustment. These are crude associations that largely parallel the age distribution of the findings (Table 3) and should not be interpreted as indications for, or outcomes of, treatment (Table 5).

## 4. Discussion

In this retrospective cross-sectional study of 496 patients with meniscal tears reported on knee MRI at a tertiary care center in Riyadh, the study population was predominantly male (65.7%) and middle-aged, and a traumatic mechanism was documented in 60.3% of patients. The medial meniscus was involved in 83.7% of patients, most often in isolation, and horizontal tears were the most frequently reported pattern, followed by radial and root tears. Joint effusion, ACL tear, and cartilage loss were the most common associated findings. Two broad clinical-imaging profiles were evident. Traumatic presentations occurred in younger, predominantly male patients and were associated with isolated lateral meniscal involvement; horizontal, vertical, bucket-handle, and complex tears; and ACL tears. Degenerative presentations occurred in older patients, more often women, and were associated with radial, root, and degenerative-pattern tears, cartilage loss, and joint effusion. These associations were robust to FDR adjustment, whereas associations involving rare findings (PCL, LCL, and MCL tears) were based on small numbers and should be interpreted cautiously. The predominance of medial meniscal involvement is consistent with large epidemiological and surgical series [3,4] and with the anatomical and biomechanical characteristics of the medial meniscus, whose capsular and deep MCL attachments limit its excursion during flexion and rotation and expose it to greater shear loading [14,15,30,31]. Conversely, isolated lateral meniscal tears were concentrated in younger patients and in traumatic cases, consistent with reports that lateral meniscal tears are relatively more frequent in acute knee injuries and in acute ACL rupture, whereas medial tears predominate in chronic ACL deficiency and in degenerative knees [10,16]. In a previous Saudi series restricted to patients with ACL injury, medial and lateral meniscal injuries were present in 49.3% and 16.2% of patients, respectively [24]; the present data extend these observations to a consecutive population of patients with MRI-reported meniscal tears.

The most notable difference from published series concerns horizontal tears. Horizontal cleavage tears are classically regarded as degenerative lesions of middle-aged and older individuals [11,32]; in our sample, however, 83.7% of horizontal tears occurred in patients with a documented traumatic mechanism, and their frequency declined with age. Several explanations are plausible. First, because tear patterns were extracted from report wording, horizontal components of acute tears in younger patients may have been reported as “horizontal”, whereas horizontal cleavage in degenerated menisci of older patients may have been reported under the broader “degenerative tear” descriptor, which accounted for 43.7% of degenerative cases. Second, the mechanism was assigned from the documented history, and minor episodes of trauma may have been recorded as the cause of symptoms in knees with pre-existing degeneration. Third, referral patterns at an institution serving a young, physically active military population may enrich for traumatic presentations. This finding should therefore be regarded as hypothesis-generating rather than as evidence of a different biological behavior of horizontal tears, and it warrants confirmation by standardized image re-reading. Because this association runs counter to the conventional morphological expectation, it is unlikely to have been produced by MRI morphology driving the mechanism classification; in contrast, the complete or near-complete separation of the complex traumatic, vertical, and degenerative categories by mechanism probably reflects the overlap between these descriptors and the mechanism variable. The associations of female sex with radial and root tears, degenerative-pattern tears, and cartilage loss accord with imaging studies showing that degenerative root tears occur predominantly in middle-aged and older women and are typically radial tears of the posterior horn of the medial meniscus, frequently with meniscal extrusion [13,33]. In our sample, however, female patients were a median of 13 years older than male patients and were more likely to have a degenerative mechanism, so these sex differences largely mirror the age and mechanism gradients and cannot be attributed to sex independently of age. Similarly, the male predominance of traumatic tears and ACL injuries agrees with earlier epidemiological data [3,4] and with the characteristics of patients with traumatic root tears [34] and probably reflects differences in age and in exposure to sports and occupational injury.

ACL tears were present in 36.1% of patients and were strongly associated with a traumatic mechanism and younger age, consistent with the frequent coexistence of meniscal and ACL injuries in rotational knee trauma [10,35]. Cartilage loss showed the opposite profile and was associated with older age and degenerative injuries, in line with systematic evidence that meniscal tears and extrusion are associated with cartilage loss and progression to joint replacement independently of age, sex, and body mass index [7,36,37]. Two findings deserve comment. Joint effusion was more frequent in degenerative cases and older patients than in traumatic cases, which may reflect the high prevalence of effusion and synovitis in osteoarthritic knees [21] and the variable interval between injury and MRI in traumatic cases. Bone contusion did not differ by mechanism and showed a non-linear age distribution; in report-based data, subchondral bone marrow edema-like lesions in degenerative knees may have been recorded as contusion, which limits the specificity of this variable [21,35].

The management patterns require particularly cautious interpretation. The proportion of surgically managed patients was higher among older patients and among those with degenerative tears or cartilage loss and lower among patients with bucket-handle or ACL tears. These patterns run counter to current recommendations, which favor early repair of displaced and unstable traumatic tears and meniscal preservation whenever possible [9,38] and do not support arthroscopic partial meniscectomy as first-line treatment for degenerative lesions [11,39]. Several features of the data are likely to contribute. Management was recorded as a binary patient-level variable, and “surgical” included any procedure on the index knee, which in older patients with cartilage loss may have been performed for associated pathology rather than for the meniscal tear itself. Conversely, “conservative” denoted the absence of a documented procedure and may include patients awaiting surgery, patients treated at other facilities, and patients who declined surgery. The specific procedure, its indication, and its timing were not available. These data therefore describe recorded management at a single institution and must not be interpreted as evidence regarding the indications for, or the effectiveness of, any treatment.

Although several of the main associations—medial predominance, the link between ACL injury and trauma, and the association between degenerative meniscal disease and cartilage loss—are well established, this study adds to the literature in three respects. First, it provides, to our knowledge, the first large consecutive MRI-based description from Saudi Arabia that simultaneously reports tear location, tear pattern, associated intra-articular abnormalities, and recorded management, stratified by age, sex, and mechanism, with effect sizes and correction for multiple testing; these data may serve as a local reference for MRI reporting and for planning musculoskeletal imaging and orthopedic services. Second, it identifies findings that differ from published series—particularly the predominance of horizontal tears among younger patients with traumatic injuries and the distribution of recorded management—which generate specific hypotheses for prospective studies. Third, it illustrates the limitations of non-standardized report descriptors for research and supports the adoption of structured knee MRI reporting with a standardized tear classification.

### Limitations and Future Directions

This study has several limitations. First, it was a retrospective, single-center study of patients referred for knee MRI at a tertiary hospital that predominantly serves National Guard personnel and their families; the findings therefore reflect a selected, hospital-based population and are subject to referral and selection bias, and the reported frequencies should not be interpreted as estimates of the incidence or prevalence of meniscal tears in the general Saudi population. Second, data were extracted from original clinical reports rather than from standardized re-reading of the images; the readers were not blinded to the clinical history, interobserver and intraobserver agreement could not be assessed, and tear patterns were recorded using descriptive categories that are not mutually exclusive and do not correspond to a standardized classification such as the ISAKOS system. Arthroscopic confirmation was not available for all patients. Third, the mechanism of injury was based on the documented history and may have incorporated imaging features when the history was incomplete, which introduces a risk of incorporation bias in the mechanism-stratified analyses. Fourth, management was recorded as a binary variable without information on procedure type, indication, timing, or clinical outcomes, and no follow-up data were available to relate tear characteristics to outcomes such as osteoarthritis progression or surgical failure. Fifth, the analyses were univariable; although effect sizes and FDR-adjusted *p*-values are reported, residual confounding, particularly by age in the sex-stratified analyses, cannot be excluded, and estimates for rare findings were imprecise. Finally, potentially relevant variables, including body mass index, activity level, occupation, symptom duration, and radiographic osteoarthritis grade, were not available. Future prospective multicenter studies should use structured MRI reporting with a standardized classification, independent double reading with assessment of reliability, predefined definitions of injury mechanism, multivariable modeling, and longitudinal clinical and patient-reported outcomes.

## 5. Conclusions

In this single-center, hospital-based sample of patients with MRI-reported meniscal tears in Riyadh, Saudi Arabia, the medial meniscus was most frequently involved, and horizontal tears were the most frequently reported pattern. Traumatic and degenerative presentations showed distinct age, sex, morphological, and associated MRI profiles: traumatic cases were younger, predominantly male, and more often had lateral meniscal, horizontal, vertical, bucket-handle, and complex tears with concomitant ACL injury, whereas degenerative cases were older, more often female, and more often had radial, root, and degenerative-pattern tears with cartilage loss. These findings are descriptive and associative, derive from a selected referral population and from original radiology reports, and should not be extrapolated to the general Saudi population or used to infer treatment effectiveness. They provide a local reference for knee MRI interpretation and generate hypotheses for prospective multicenter studies using standardized classification and outcome assessment.

## Figures and Tables

**Table 1:** Demographic and clinical characteristics of the 496 patients with meniscal tears.

Characteristic	*n* (%)	95% CI
**Sex**		
Male	326 (65.7)	61.4–69.8
Female	170 (34.3)	30.2–38.6
**Age, years**		
Mean ± SD	41.78 ± 13.21	
Median (IQR)	40 (20)	
Range (min-max)	15–87	
**Age group, years**		
≤25	47 (9.5)	7.2–12.4
26–35	133 (26.8)	23.1–30.9
36–45	129 (26.0)	22.3–30.0
46–55	98 (19.8)	16.5–23.5
56–65	69 (13.9)	11.1–17.2
>65	20 (4.0)	2.6–6.1
**Mechanism of injury**		
Traumatic	299 (60.3)	55.9–64.5
Degenerative	197 (39.7)	35.5–44.1
**Tear location**		
Isolated medial meniscus	344 (69.4)	65.2–73.3
Isolated lateral meniscus	81 (16.3)	13.3–19.8
Both menisci	71 (14.3)	11.5–17.7
Any medial meniscal involvement	415 (83.7)	80.2–86.7
Any lateral meniscal involvement	152 (30.6)	26.7–34.8
**Tear pattern ‡**		
Radial	97 (19.6)	16.3–23.3
Horizontal	135 (27.2)	23.5–31.3
Root	96 (19.4)	16.1–23.1
Bucket-handle	24 (4.8)	3.3–7.1
Complex traumatic	77 (15.5)	12.6–19.0
Degenerative	90 (18.1)	15.0–21.8
Vertical	28 (5.6)	3.9–8.0
**Associated findings ‡**		
None	85 (17.1)	14.1–20.7
ACL tear	179 (36.1)	32.0–40.4
PCL tear	7 (1.4)	0.7–2.9
Cartilage loss	170 (34.3)	30.2–38.6
Bone contusion	128 (25.8)	22.2–29.8
Joint effusion	291 (58.7)	54.3–62.9
LCL tear	9 (1.8)	1.0–3.4
MCL tear	16 (3.2)	2.0–5.2
**Management**		
Surgical	333 (67.1)	62.9–71.1
Conservative	163 (32.9)	28.9–37.1

Percentages are based on the total sample (*n* = 496). 95% CI, Wilson confidence interval for the proportion. ‡ Multiple-response variables: more than one tear pattern (547 patterns in 496 patients; percentages sum to 110.3%) and more than one associated finding could be recorded in the same patient; therefore, percentages do not sum to 100%. ACL, anterior cruciate ligament; IQR, interquartile range; LCL, lateral collateral ligament; MCL, medial collateral ligament; PCL, posterior cruciate ligament; SD, standard deviation.

**Table 2:** Demographic and clinical characteristics of patients with meniscal tears according to sex.

Characteristic	Male (*n* = 326)	Female (*n* = 170)	*p*	q (FDR)	OR (95% CI) or V
Age, years, median (IQR)	36 (15)	49 (17)	<0.001	<0.001	—
**Age group, years**					
≤25	43 (13.2)	4 (2.4)	<0.001	<0.001	V = 0.43
26–35	108 (33.1)	25 (14.7)			
36–45	99 (30.4)	30 (17.6)			
46–55	37 (11.3)	61 (35.9)			
56–65	26 (8.0)	43 (25.3)			
>65	13 (4.0)	7 (4.1)			
**Mechanism of injury**					
Traumatic	240 (73.6)	59 (34.7)	<0.001	<0.001	5.25 (3.52–7.84)
Degenerative	86 (26.4)	111 (65.3)			
**Tear location**					
Isolated medial meniscus	213 (65.3)	131 (77.1)	0.017	0.034	V = 0.13
Isolated lateral meniscus	63 (19.3)	18 (10.6)			
Both menisci	50 (15.3)	21 (12.4)			
**Tear pattern ‡**					
Radial	46 (14.1)	51 (30.0)	<0.001	<0.001	0.38 (0.24–0.60)
Horizontal	97 (29.8)	38 (22.4)	0.079	0.105	1.47 (0.96–2.27)
Root	54 (16.6)	42 (24.7)	0.029	0.045	0.61 (0.38–0.95)
Bucket-handle	24 (7.4)	0 (0.0)	<0.001	<0.001	NE
Complex traumatic	68 (20.9)	9 (5.3)	<0.001	<0.001	4.71 (2.29–9.71)
Degenerative	50 (15.3)	40 (23.5)	0.025	0.042	0.59 (0.37–0.94)
Vertical	24 (7.4)	4 (2.4)	0.022	0.040	3.30 (1.13–9.67)
**Associated findings ‡**					
None	58 (17.8)	27 (15.9)	0.592	0.658	1.15 (0.70–1.89)
ACL tear	159 (48.8)	20 (11.8)	<0.001	<0.001	7.14 (4.27–11.95)
PCL tear *	2 (0.6)	5 (2.9)	0.050	0.071	0.20 (0.04–1.06)
Cartilage loss	73 (22.4)	97 (57.1)	<0.001	<0.001	0.22 (0.15–0.32)
Bone contusion	85 (26.1)	43 (25.3)	0.851	0.851	1.04 (0.68–1.59)
Joint effusion	186 (57.1)	105 (61.8)	0.312	0.367	0.82 (0.56–1.20)
LCL tear *	8 (2.5)	1 (0.6)	0.175	0.219	4.25 (0.53–34.28)
MCL tear	11 (3.4)	5 (2.9)	0.796	0.838	1.15 (0.39–3.37)
**Management**					
Surgical	186 (57.1)	147 (86.5)	<0.001	<0.001	0.21 (0.13–0.34)
Conservative	140 (42.9)	23 (13.5)			

Values are *n* (% of the column group) unless otherwise stated. OR, unadjusted odds ratio for males vs. females (reference); *p*, unadjusted *p*-value (Pearson’s chi-square test unless otherwise indicated; age compared using the Mann–Whitney U test); * Fisher’s exact test; q, Benjamini–Hochberg false discovery rate (FDR)-adjusted *p*-value within this table; V, Cramér’s V for multicategory variables; NE, not estimable (zero cell). ‡ Multiple-response variables; each category was tested as present vs. absent. ACL, anterior cruciate ligament; IQR, interquartile range; LCL, lateral collateral ligament; MCL, medial collateral ligament; PCL, posterior cruciate ligament.

**Table 3:** Demographic and clinical characteristics of patients with meniscal tears according to age group (years).

Characteristic	≤25 (*n* = 47)	26–35 (*n* = 133)	36–45 (*n* = 129)	46–55 (*n* = 98)	56–65 (*n* = 69)	>65 (*n* = 20)	*p*	q (FDR)	V
**Mechanism of injury**									
Traumatic	46 (97.9)	123 (92.5)	79 (61.2)	25 (25.5)	22 (31.9)	4 (20.0)	<0.001	<0.001	0.59
Degenerative	1 (2.1)	10 (7.5)	50 (38.8)	73 (74.5)	47 (68.1)	16 (80.0)			
**Sex**									
Male	43 (91.5)	108 (81.2)	99 (76.7)	37 (37.8)	26 (37.7)	13 (65.0)	<0.001	<0.001	0.43
Female	4 (8.5)	25 (18.8)	30 (23.3)	61 (62.2)	43 (62.3)	7 (35.0)			
**Tear location**									
Isolated medial meniscus	25 (53.2)	76 (57.1)	94 (72.9)	82 (83.7)	52 (75.4)	15 (75.0)	<0.001	<0.001	0.21
Isolated lateral meniscus	13 (27.7)	37 (27.8)	23 (17.8)	3 (3.1)	5 (7.2)	0 (0.0)			
Both menisci	9 (19.1)	20 (15.0)	12 (9.3)	13 (13.3)	12 (17.4)	5 (25.0)			
**Tear pattern ‡**									
Radial	2 (4.3)	17 (12.8)	22 (17.1)	33 (33.7)	18 (26.1)	5 (25.0)	<0.001	<0.001	0.23
Horizontal	19 (40.4)	50 (37.6)	38 (29.5)	12 (12.2)	12 (17.4)	4 (20.0)	<0.001	<0.001	0.23
Root	7 (14.9)	15 (11.3)	23 (17.8)	26 (26.5)	24 (34.8)	1 (5.0)	<0.001	<0.001	0.21
Bucket-handle *	6 (12.8)	11 (8.3)	7 (5.4)	0 (0.0)	0 (0.0)	0 (0.0)	<0.001	0.001	0.20
Complex traumatic	11 (23.4)	37 (27.8)	16 (12.4)	8 (8.2)	3 (4.3)	2 (10.0)	<0.001	<0.001	0.24
Degenerative	2 (4.3)	7 (5.3)	28 (21.7)	26 (26.5)	19 (27.5)	8 (40.0)	<0.001	<0.001	0.27
Vertical *	7 (14.9)	12 (9.0)	6 (4.7)	1 (1.0)	2 (2.9)	0 (0.0)	0.008	0.011	0.18
**Associated findings ‡**									
None	8 (17.0)	27 (20.3)	29 (22.5)	12 (12.2)	7 (10.1)	2 (10.0)	0.142	0.150	0.13
ACL tear	32 (68.1)	79 (59.4)	43 (33.3)	8 (8.2)	12 (17.4)	5 (25.0)	<0.001	<0.001	0.44
PCL tear *	1 (2.1)	0 (0.0)	1 (0.8)	2 (2.0)	3 (4.3)	0 (0.0)	0.133	0.149	0.12
Cartilage loss	6 (12.8)	12 (9.0)	41 (31.8)	59 (60.2)	39 (56.5)	13 (65.0)	<0.001	<0.001	0.45
Bone contusion	19 (40.4)	23 (17.3)	28 (21.7)	31 (31.6)	19 (27.5)	8 (40.0)	0.009	0.011	0.18
Joint effusion	26 (55.3)	61 (45.9)	72 (55.8)	72 (73.5)	45 (65.2)	15 (75.0)	0.001	0.001	0.21
LCL tear *	0 (0.0)	7 (5.3)	0 (0.0)	2 (2.0)	0 (0.0)	0 (0.0)	0.030	0.036	0.17
MCL tear *	1 (2.1)	8 (6.0)	1 (0.8)	2 (2.0)	3 (4.3)	1 (5.0)	0.167	0.167	0.12
**Management**									
Surgical	19 (40.4)	65 (48.9)	88 (68.2)	80 (81.6)	63 (91.3)	18 (90.0)	<0.001	<0.001	0.37
Conservative	28 (59.6)	68 (51.1)	41 (31.8)	18 (18.4)	6 (8.7)	2 (10.0)			

Values are *n* (% of the age group, i.e., column percentages). *p*, unadjusted *p*-value (Pearson’s chi-square test unless otherwise indicated); * Fisher’s exact test; q, Benjamini–Hochberg false discovery rate (FDR)-adjusted *p*-value within this table; V, Cramér’s V. ‡ Multiple-response variables; each category was tested as present vs. absent across age groups. Age was not tested because it is the stratifying variable. ACL, anterior cruciate ligament; LCL, lateral collateral ligament; MCL, medial collateral ligament; PCL, posterior cruciate ligament.

**Table 4:** Demographic and clinical characteristics of patients with meniscal tears according to mechanism of injury.

Characteristic	Traumatic (*n* = 299)	Degenerative (*n* = 197)	*p*	q (FDR)	OR (95% CI) or V
Age, years, median (IQR)	34 (13)	51 (15)	<0.001	<0.001	—
**Sex**					
Male	240 (80.3)	86 (43.7)	<0.001	<0.001	5.25 (3.52–7.84)
Female	59 (19.7)	111 (56.3)			
**Tear location**					
Isolated medial meniscus	191 (63.9)	153 (77.7)	<0.001	<0.001	V = 0.21
Isolated lateral meniscus	68 (22.7)	13 (6.6)			
Both menisci	40 (13.4)	31 (15.7)			
**Tear pattern ‡**					
Radial	38 (12.7)	59 (29.9)	<0.001	<0.001	0.34 (0.22–0.54)
Horizontal	113 (37.8)	22 (11.2)	<0.001	<0.001	4.83 (2.93–7.98)
Root	47 (15.7)	49 (24.9)	0.012	0.015	0.56 (0.36–0.88)
Bucket-handle	23 (7.7)	1 (0.5)	<0.001	<0.001	16.33 (2.19–121.96)
Complex traumatic	77 (25.8)	0 (0.0)	<0.001	<0.001	NE
Degenerative	4 (1.3)	86 (43.7)	<0.001	<0.001	0.02 (0.01–0.05)
Vertical	28 (9.4)	0 (0.0)	<0.001	<0.001	NE
**Associated findings ‡**					
None	62 (20.7)	23 (11.7)	0.009	0.012	1.98 (1.18–3.32)
ACL tear	147 (49.2)	32 (16.2)	<0.001	<0.001	4.99 (3.21–7.75)
PCL tear *	1 (0.3)	6 (3.0)	0.017	0.020	0.11 (0.01–0.89)
Cartilage loss	48 (16.1)	122 (61.9)	<0.001	<0.001	0.12 (0.08–0.18)
Bone contusion	70 (23.4)	58 (29.4)	0.133	0.140	0.73 (0.49–1.10)
Joint effusion	159 (53.2)	132 (67.0)	0.002	0.003	0.56 (0.38–0.81)
LCL tear *	7 (2.3)	2 (1.0)	0.329	0.329	2.34 (0.48–11.37)
MCL tear	14 (4.7)	2 (1.0)	0.029	0.032	4.79 (1.08–21.31)
**Management**					
Surgical	163 (54.5)	170 (86.3)	<0.001	<0.001	0.19 (0.12–0.30)
Conservative	136 (45.5)	27 (13.7)			

Values are *n* (% of the column group) unless otherwise stated. OR, unadjusted odds ratio for traumatic vs. degenerative (reference) cases; *p*, unadjusted *p*-value (Pearson’s chi-square test unless otherwise indicated; age compared using the Mann–Whitney U test); * Fisher’s exact test; q, Benjamini–Hochberg false discovery rate (FDR)-adjusted *p*-value within this table; V, Cramér’s V for multicategory variables; NE, not estimable (zero cell). ‡ Multiple-response variables; each category was tested as present vs. absent. ACL, anterior cruciate ligament; IQR, interquartile range; LCL, lateral collateral ligament; MCL, medial collateral ligament; PCL, posterior cruciate ligament. The tear-pattern descriptors “complex traumatic” and “degenerative” partly overlap with the mechanism variable (see Section 2.6).

**Table 5:** Recorded management according to tear pattern and associated findings.

Characteristic (*n* with Finding)	Surgical (*n* = 333)	Conservative (*n* = 163)	*p*	q (FDR)	OR (95% CI)
**Tear pattern ‡**					
Radial (*n* = 97)	75 (77.3)	22 (22.7)	0.017	0.043	1.86 (1.11–3.13)
Horizontal (*n* = 135)	86 (63.7)	49 (36.3)	0.319	0.399	0.81 (0.53–1.23)
Root (*n* = 96)	67 (69.8)	29 (30.2)	0.537	0.575	1.16 (0.72–1.89)
Bucket-handle (*n* = 24)	4 (16.7)	20 (83.3)	<0.001	<0.001	0.09 (0.03–0.26)
Complex traumatic (*n* = 77)	33 (42.9)	44 (57.1)	<0.001	<0.001	0.30 (0.18–0.49)
Degenerative (*n* = 90)	81 (90.0)	9 (10.0)	<0.001	<0.001	5.50 (2.68–11.27)
Vertical (*n* = 28)	17 (60.7)	11 (39.3)	0.456	0.526	0.74 (0.34–1.63)
**Associated findings ‡**					
None (*n* = 85)	66 (77.6)	19 (22.4)	0.023	0.049	1.87 (1.08–3.24)
ACL tear (*n* = 179)	77 (43.0)	102 (57.0)	<0.001	<0.001	0.18 (0.12–0.27)
PCL tear * (*n* = 7)	7 (100.0)	0 (0.0)	0.102	0.191	NE
Cartilage loss (*n* = 170)	139 (81.8)	31 (18.2)	<0.001	<0.001	3.05 (1.95–4.77)
Bone contusion (*n* = 128)	91 (71.1)	37 (28.9)	0.269	0.367	1.28 (0.83–1.99)
Joint effusion (*n* = 291)	194 (66.7)	97 (33.3)	0.790	0.790	0.95 (0.65–1.39)
LCL tear * (*n* = 9)	4 (44.4)	5 (55.6)	0.162	0.243	0.38 (0.10–1.45)
MCL tear (*n* = 16)	8 (50.0)	8 (50.0)	0.138	0.230	0.48 (0.18–1.29)

Values are *n* (% of patients with the finding, i.e., row percentages). OR, unadjusted odds ratio for surgical management in patients with vs. without the finding. Surgical management: any arthroscopic or open procedure on the index knee documented after the index MRI, irrespective of whether it addressed the meniscal tear or an associated lesion; conservative management: no documented surgical procedure. *p*, unadjusted *p*-value (Pearson’s chi-square test unless otherwise indicated); * Fisher’s exact test; q, Benjamini–Hochberg false discovery rate (FDR)-adjusted *p*-value within this table; NE, not estimable (zero cell). ‡ Multiple-response variables. ACL, anterior cruciate ligament; LCL, lateral collateral ligament; MCL, medial collateral ligament; PCL, posterior cruciate ligament.

## Data Availability

The data presented in this study are available on request from the corresponding author.

## Supplementary Materials

The following supporting information can be downloaded at: https://www.mdpi.com/article/10.3390/diagnostics16213650/s1, Table S1: STROBE checklist for cross-sectional studies.
