# Supplementary material for: MRI-Based Patterns of Meniscal Tears and Associated Intra-Articular Abnormalities in Patients at a Tertiary Care Center in Riyadh, Saudi Arabia: A Retrospective Cross-Sectional Study
Source: Diagnostics (Basel). 2026 Nov 4;16(21):3650. doi: 10.3390/diagnostics16213650 (PMC13761737; doi:10.3390/diagnostics16213650)
Supplement: Supplementary file 1 [file diagnostics-16-03650-s001.zip › diagnostics-4638894-supplementary.pdf]

Supplementary Table S1. STROBE checklist for cross-sectional studies

MRI-Based Patterns of Meniscal Tears and Associated Intra-Articular Abnormalities in Patients at a Tertiary Care Center in Riyadh, Saudi Arabia: A Retrospective Cross-Sectional Study

Section/Topic	Item No.	Recommendation (cross-sectional studies)	Location in revised manuscript
**Title and abstract**	1	(a) Indicate the study's design with a commonly used term in the title or the abstract	Title; Abstract (Methods)
		(b) Provide in the abstract an informative and balanced summary of what was done and what was found	Abstract
**Introduction**			
**Background/rationale**	2	Explain the scientific background and rationale for the investigation being reported	Introduction, paragraphs 1-4
**Objectives**	3	State specific objectives, including any prespecified hypotheses	Introduction, paragraph 4
**Methods**			
**Study design**	4	Present key elements of study design early in the paper	Section 2.1
**Setting**	5	Describe the setting, locations, and relevant dates, including periods of recruitment, exposure, follow-up, and data collection	Sections 2.2 and 2.3
**Participants**	6	(a) Give the eligibility criteria, and the sources and methods of selection of participants	Section 2.3; Figure 1
**Variables**	7	Clearly define all outcomes, exposures, predictors, potential confounders, and effect modifiers. Give diagnostic criteria, if applicable	Sections 2.5 and 2.6
**Data sources/measurement**	8	For each variable of interest, give sources of data and details of methods of assessment (measurement)	Sections 2.4-2.6
**Bias**	9	Describe any efforts to address potential sources of bias	Sections 2.3, 2.5, 2.6; Section 4.1
**Study size**	10	Explain how the study size was arrived at	Section 2.3 (all consecutive eligible patients); Figure 1
**Quantitative variables**	11	Explain how quantitative variables were handled in the analyses	Section 2.7 (age as continuous and in six groups)
**Statistical methods**	12	(a) Describe all statistical methods, including those used to control for confounding	Section 2.7
		(b) Describe any methods used to examine subgroups and interactions	Section 2.7 (stratification by sex, age group, mechanism, management)
		(c) Explain how missing data were addressed	Section 2.7 (no missing data; complete data were an inclusion criterion)
		(d) If applicable, describe analytical methods taking account of sampling strategy	Not applicable (consecutive single-center sample)
		(e) Describe any sensitivity analyses	Section 2.7 (Benjamini-Hochberg FDR adjustment)
**Results**			
**Participants**	13	(a) Report numbers of individuals at each stage of study	Section 3.1; Figure 1 (source population and final sample, n = 496)
		(b) Give reasons for non-participation at each stage	Section 2.3; Figure 1 (predefined exclusion criteria)
		(c) Consider use of a flow diagram	Figure 1

**Descriptive data**	14	(a) Give characteristics of study participants and information on exposures and potential confounders	Section 3.1; Table 1
		(b) Indicate number of participants with missing data for each variable of interest	Section 2.7 (none)
**Outcome data**	15	Report numbers of outcome events or summary measures	Sections 3.1-3.5; Tables 1-5
**Main results**	16	(a) Give unadjusted estimates and, if applicable, confounder-adjusted estimates and their precision (e.g., 95% CI)	Sections 3.2-3.5; Tables 2-5 (unadjusted ORs with 95% CIs)
		(b) Report category boundaries when continuous variables were categorized	Table 1; Table 3
		(c) If relevant, consider translating estimates of relative risk into absolute risk	Not applicable
**Other analyses**	17	Report other analyses done (e.g., subgroups, interactions, sensitivity analyses)	Tables 2-5 (FDR-adjusted q-values); Figure 2
**Discussion**			
**Key results**	18	Summarize key results with reference to study objectives	Section 4, paragraph 1
**Limitations**	19	Discuss limitations of the study, taking into account sources of potential bias or imprecision; discuss direction and magnitude of any potential bias	Section 4.1
**Interpretation**	20	Give a cautious overall interpretation of results considering objectives, limitations, multiplicity of analyses, results from similar studies, and other relevant evidence	Section 4, paragraphs 2-7; Section 5
**Generalisability**	21	Discuss the generalisability (external validity) of the study results	Section 4.1; Section 5
**Other information**			
**Funding**	22	Give the source of funding and the role of the funders	Funding statement

Checklist items adapted from von Elm E, et al. The Strengthening the Reporting of Observational Studies in Epidemiology (STROBE) statement. Lancet 2007;370:1453-1457.
